# Labour and social protection gaps impacting the health and well-being of workers in non-standard employment: An international comparative study

**DOI:** 10.1371/journal.pone.0320248

**Published:** 2025-03-25

**Authors:** Signild Kvart, Isabel Cuervo, Virginia Gunn, Wayne Lewchuk, Kim Bosmans, Letitia Davis, Astrid Escrig-Piñol, Per-Olof Östergren, Eva Padrosa, Alejandra Vives, Alessandro Zaupa, Emily Q. Ahonen, Valentina Álvarez-López, Mireia Bolíbar, Ignacio Diaz, Mariana Gutiérrez-Zamora, Lars Ivarsson, Mireia Julià, Carles Muntaner, Patricia O’Campo, Marisol E. Ruiz, Kristian Vänerhagen, Emilia F. Vignola, David Vílchez, Mattias Vos, Theo Bodin, Sherry L. Baron

**Affiliations:** 1 Unit of Occupational Medicine, Institute of Environmental Medicine, Karolinska Institutet, Stockholm, Sweden; 2 Barry Commoner Center for Health and the Environment, Queens College, City University of New York, New York, United States of America; 3 MAP Centre for Urban Health Solutions, Li Ka Shing Knowledge Institute, Unity Health Toronto, Toronto, Ontario, Canada; 4 School of Nursing, Cape Breton University, Sydney, Canada; 5 Department of Economics and School of Labour Studies, McMaster University, Hamilton, Canada; 6 Department of Sociology, Brussels Institute for Social and Population Studies, Vrije Universiteit Brussel, Brussels, Belgium; 7 Independent researcher, Cambridge, Massachusetts, United States of America; 8 Social Determinants and Health Education Research Group (SDHEd), Hospital del Mar Research Institute, Barcelona, Spain; 9 Hospital del Mar Nursing School (ESIHMar), Universitat Pompeu Fabra-affiliated, Barcelona, Spain; 10 Social Medicine and Global Health, Department of Clinical Sciences Malmö, Lund University, Malmö, Sweden; 11 Research Group on Health Inequalities, Environment - Employment Conditions (GREDS-EMCONET), Department of Political and Social Sciences, Universitat Pompeu Fabra, Barcelona, Spain; 12 School of Public Health, School of Medicine, Pontificia Universidad Católica de Chile, Santiago, Chile; 13 Center for Sustainable Urban Development, CEDEUS, Pontificia Universidad Católica de Chile, Santiago, Chile; 14 Department of Family and Preventive Medicine, Division of Occupational and Environmental Health, University of Utah School of Medicine, Salt Lake City, Utah, United States of America; 15 Department of Territorial Studies and Intercultural Dialogues, Universidad de Playa Ancha, Valparaíso, Chile; 16 Centre d’Estudis Sociològics sobre la Vida Quotidiana i el Treball (QUIT), Department of Sociology, Universitat Autònoma de Barcelona, Cerdanyola del Vallès, Spain; 17 Johns Hopkins University—Universitat Pompeu Fabra Public Policy Center, UPF Barcelona School of Management, Universitat Pompeu Fabra, Barcelona, Spain; 18 Karlstad Business School, Karlstad University, Karlstad, Sweden; 19 Lawrence S. Bloomberg Faculty of Nursing, St. George Campus, Toronto, Ontario, Canada; 20 Institute of Public Health, School of Medicine, Universidad Austral de Chile, Valdivia, Chile; 21 Department of Community Health and Social Sciences, City University of New York Graduate School of Public Health and Health Policy, New York, United States of America; 22 Center for Occupational and Environmental Medicine, Stockholm Region, Stockholm, Sweden; Wroclaw University of Economics and Business: Uniwersytet Ekonomiczny we Wroclawiu, POLAND

## Abstract

**Background:**

World economies increasingly rely on non-standard employment arrangements, which has been linked to ill health. While work and employment conditions are recognized structural determinants of health and health equity, policies aiming to protect workers from negative implications predominantly focus on standard employment arrangements and the needs of workers in non-standard employment may be neglected. The aim of this study is to explore workers’ experiences of gaps in labour regulations and social protections and its influence on their health and well-being across 6 countries with differing policy approaches: Belgium, Canada, Chile, Spain, Sweden, and the United States.

**Methods:**

250 semi-structured interviews with workers in non-standard employment were analyzed thematically using a multiple case-study approach.

**Results:**

There are notable differences in workers’ rights to protection across the countries. However, participants across all countries experienced similar challenges including employment instability, income inadequacy and limited rights and protection, due to policy-related gaps and access-barriers. In response, they resorted to individual resources and strategies, struggled to envision supportive policies, and expressed low expectations of changes by employers and policymakers.

**Conclusions:**

Policy gaps threaten workers’ health and well-being across all study countries, irrespective of the levels of labour market regulations and social protections. Workers in non-standard employment disproportionately endure economic risks, which may increase social and health inequality. The study highlights the need to improve social protection for this vulnerable population.

## Introduction

World economies continue to move towards competitive market-based practices driven by increased globalization, technological progress, international trade agreements, demographic trends, and the changing nature of work [1,2]. Such market-based practices have complex implications for population health and well-being including the impact of these practices on non-standard employment (NSE) arrangements. NSE is a departure from standard employment understood as “work that is full time, indefinite, as well as part of a subordinate relationship between an employee and an employer” [3]. It encompasses a variety of work arrangements including part-time, temporary, seasonal or on-call work, day labour, employment through web-based platforms and self-employment [3].

Understanding the health and well-being consequences of NSE is crucial in assessing the worker implications of labour market flexibility. NSE can be beneficial in providing positive work experiences and expanded networks and may in some cases be preferred over standard employment, for instance among people with caretaking responsibilities [3]. Qualitative research has indicated that NSE workers sometimes perceive greater control over their careers and enjoy the flexibility that NSE can offer, which is associated with improved mental health and well-being [4,5]. On the other hand, NSE is most often associated with a degree of precariousness, which could negatively impact the health and well-being of individuals and their families [6–11].

Precarious Employment (PE) is a multidimensional construct in which components of the employment relationship, including length or type of contract (i.e., degree of employment instability), inadequate levels of pay and other non-wage benefits and (lack of) rights, protection and representation, are considered to negatively affect workers’ health and well-being [12,13]. Pathways linking PE (common to NSE arrangements) to health and well-being include material deprivation, workplace hazards, and negative psychological effects related to insecurity, unpredictable scheduling and revenues, or lack of protection [12,14]. PE has been linked to various cardiovascular [8] and mental health disorders [15], occupational accidents and injuries [16], diminished well-being [17], and harmful lifestyle behaviors [18], and are thus important determinants of population health and health inequities not only nationally but globally [19–21]. NSE, when precarious, can therefore generate multiple insecurities among workers, especially when it is not voluntary or only undertaken because the worker’s life situation necessitates the flexibility NSE offers [3,22]. These health burdens are likely to compound existing health inequalities as women and migrants are disproportionately employed in more precarious forms of NSE and more often earn lower wages compared to workers in standard employment [3].

Previous research has highlighted the importance of employment as one of the social determinants of health and the need for policy solutions to reduce health inequities arising from it [19,23]. Some studies have indicated that labour and social policies can buffer the negative impacts of NSE [24–31]. Other studies however have not found this association [32,33]. The mixed findings may be contributed to distinct policy challenges facing NSE workers and potential gaps in their coverage under existing labour and social policies [34–37]. Despite the proliferation of NSE, regulatory frameworks have been slow to adapt, and the predominant focus of labour regulations and social protection policies has remained on workers in standard employment arrangements [38–41]. This neglect may create gaps in regulations and policies resulting in unmet needs among workers in NSE, further affecting their health and well-being [38]. Ways in which varying regulatory contexts and existing labour and social policies influence the challenges faced by workers in NSE are yet to be fully explored and results can provide important insights for future policy approaches that will enhance health and health equity. Therefore, this understudied area of research merits further attention.

In our study, we examine the nature of NSE drawing on the framework of PE as proposed by Bodin et al [12] and further defined by Kreshpaj et al [13]. Although not all NSE arrangements can be characterized as precarious, the PE framework includes wide ranging dimensions of key importance for health and well-being and provides a useful frame for exploring how and when NSE can be linked to health.

The aim of this study, part of a larger research program (precariousworkresearch.org), was to better understand how differing approaches to labour market regulations and social protections influence the lived experiences of workers in NSE. Our overarching focus was on understanding the ways in which workers experience the effects of their employment arrangements on their health and well-being. Specifically, we explored the following research questions:

1. How do workers in NSE describe their experiences of labour market regulations and social protection policies including:
  a. What policy-related gaps and challenges do they describe?
  b. What responses and strategies do they describe in relation to policy-related gaps and challenges?
  c. How do they envision potential solutions that could strengthen their health and well-being?
2. How can the experiences, responses and envisioned solutions be understood in relation to the differing policy contexts?

The study draws on qualitative data from workers in NSE with varying levels of precarity across six high-income countries—Belgium, Canada, Chile, Spain, Sweden, and the United States (US)—each with distinct economic and welfare state models.

### Labour regulations and social protections context of the study countries

Using the welfare regimes/ varieties of capitalism approach developed by several authors [42–46] we reviewed national (sometimes regional) labour regulations and social protection policies of the six countries included in this study. We selected 19 indicators available for all six countries and used these indicators to create summary indexes and calculated averages across the six countries (Table 1 and Supporting information S1 Table), and then compared each country’s indicators to those averages. Table 1 includes a visual representation of these indicators. Green cells generally represent more supportive policies for workers and orange cells less supportive policies. Indicator descriptions, data sources and detailed scores are included in Supporting information S1 Table. The results highlight the very different models of economic and welfare state organization of the six high-income countries. As discussed in more detail below, the United States (US) and Canada generally provide the weakest labour regulations and least generous social protections of the six countries, whereas regulations and supports are generally stronger in the other four countries.

**Table 1:** Comparison of labour market regulations and social expenditure indicators ^*^ .

Indicator	Canada	US	Chile	Sweden	Belgium	Spain
**Labour market regulation indicators**						
Labour market regulation index						
Components of the labour market regulation index						
Strictness of employment protection of regular contracts						
Strictness of employment protection of temporary agency and fixed term contracts						
Regulation of collective dismissals of regular contracts						
Regulations for hiring temporary agency contracts						
Regulations for hiring fixed term contracts						
**Other labour market indicators**						
Active labour market expenditure						
Passive labour market expenditure						
Jobless income benefits						
Paid sicks days index						
Minimum wage						
Union density: private sector						
Union density: workers with temporary contracts						
**Social protection indicators**						
Overall social expenditure						
Specific indicators of social protection						
Income support to working age pop						
Population coverage for health care						
Paid maternity, parental and home care leave available to mothers, *duration (weeks)*						
Paid maternity, parental and home care leave available to mothers, *amount (average payment rate)*						
Pension spending (public)						

*Colour codes relative to the average for 6 countries:

More than 25% above six-country average: green

Between 10-25% more than six-country average: light green

Within plus minus 10% of six-country average: white

Between 10-25% below six-country average: light brown

More than 25% below six-country average: brown

The detailed scores, measurements, and data sources are shown in Supporting information S1 Table

### Labour market regulations

Both Canada and the US stand out as having less comprehensive labour regulations than any of the other countries (Table 1). Belgium, Spain, and Chile provide the most protection to workers in temporary and fixed-term contracts. Given that available employment protection legislation indicators refer to either workers in regular employment or in temporary employment but not the self-employed, it seems that none of the countries provide a particularly comprehensive set of regulatory protection for self-employed workers.

Regarding other labour market policies, Canada, Chile, and the US provide limited support, both active and passive. Active labour market support includes policies designed to move workers into jobs when out of work or into better paying, more stable jobs while passive support is designed to replace income when workers are out of work [47]. Belgium and Spain provide both active and passive supports while Sweden emphasizes active support and provides only modest passive support. The level of jobless income support is higher in the European countries than in the Americas. Sweden and Belgium have the highest coverage of paid sick leave, followed by Spain and Chile.

Since workers in NSE are often employed in lower paying positions, higher minimum wage policies should help them [3]. Both Belgium and Canada provide relatively high minimum wage supports; Chile has the lowest minimum wage policy. Sweden does not regulate minimum wages, but follows a tripartite model where wages are stipulated in collective agreements without government involvement [48]. In the US, there is a federal minimum wage and 30 out of 50 states have set higher wages, which override the lower federal minimum wage [49].

Only Sweden and Belgium have relatively high union density levels for workers in general and for workers with fixed-term contracts. Workers in the US are least likely to be members of unions.

### Social protections

Comparison of overall social expenditures (as a percentage of Gross Domestic Product) highlights broad differences between the three European countries and the three countries in the Americas. Breaking down social protection expenditures by type, countries have adopted different strategies. Belgium, Sweden, Spain, and Canada give more priority to expenditures that support working age populations.

A major social protection gap in the US is the absence of a public healthcare system for the working population; health coverage is employer-based. Workers in the US and, to some extent Canada, face increased insecurity, as the loss of employment can also mean loss of health benefits. The other five countries provide at least basic public healthcare. European plans tend to be more comprehensive, covering most medical services, drugs, and some dental and vision care; while in Canada, drugs, dental, and vision services are generally not covered by public plans. Chile has a dual health system, with a public finance system providing coverage for a majority of the population, and a smaller private insurance system providing coverage above allowable costs. The six countries take different approaches to supporting the raising of children and retirement benefits. For instance, whereas duration of paid parental and home care leave available to mothers is the longest in Sweden, followed by Canada and Belgium, our analysis shows that Spain and Chile provide the highest level of income replacement during such paid leaves. There is no federal paid leave in the US and only a few states provide some support.

Belgium and Sweden provide the most generous public support to retired workers, while Canada, Spain, and to some degree the US, rely more heavily on retirement support provided through employment. In Chile, retired workers rely on individual savings in a private mandatory pension system.

## Materials and methods

### Study design

We chose a multi-case study design inspired by Stake [50], where the phenomenon of interest was NSE and its influences on the health and well-being of workers and their families. This approach allowed us to examine the cases (countries in this research) both separately—to study the phenomenon in its “situational uniqueness”—and together, in order to get further insight into the phenomenon itself [50].

We drew on individual worker data obtained through semi-structured interviews [51] with workers in NSE with varying levels of precariousness. The interview data brings forward the perspective of workers in NSE, capturing their experiences, perceptions, and feelings about the phenomenon.

### Ethical considerations

The study was approved by the ethical review board of Stockholm, Sweden (2020-02396). Each participating country obtained additional ethical approvals from their respective regional ethical review board. All study participants were provided written and oral information and signed a consent form prior to the interview. Ethical approval numbers: Sweden and Spain (Reg # 2020-02396, Karolinska Institutet, Stockholm County), Belgium (Ref # ECHW_228, Vrije 221 Universiteit Brussel, Brussel), US (REF # 2020-0412, Queens College, City University of New York, 223 NY), Canada (REB 20-110, MAP Centre for Urban Health Solutions, Li Ka Shing 224 Knowledge Institute, Unity Health, ON), and Chile (Reg # 2020- 012321, Pontificia Universidad Católica de Chile, Santiago). Additional information regarding the ethical, cultural, and scientific considerations specific to inclusivity in global research is included in the Supporting Information (S1 Checklist).

### Sampling and recruitment

We recruited 250 workers in NSE from: Belgium (38; whole country), Canada (40; Ontario), Chile (40; Santiago, Concepción, and Valparaíso), Spain (41; Catalonia), Sweden (51; Stockholm and Värmland), and the US (40; New York City metropolitan area), through an online questionnaire that was open between October 2020 through June 2021. In five out of the six countries we targeted workers in specific geographical areas (e.g., provinces, counties, states), rather than targeting the whole country, to limit heterogeneity in the socio-economic context. In Belgium, workers were recruited across the whole country to ensure enough representation across the varying language communities (French and Dutch). Workers were considered eligible if any of the following NSE conditions were met: (i) not being employed directly, (ii) not working full-time, (iii) not having an open-ended or permanent contract, or (iv) being in informal employment (defined as not paying taxes or without active pension contributions). Age eligibility was 25-55 to focus on workers in their prime working years and with potential family responsibilities.

Country-specific outreach methods, predominantly social media advertising, were used to reach a broad sample of this diverse and hard to reach worker population [25]. A stratified heterogeneous sample was drawn from individuals who agreed to be contacted for an interview. Strata considered were gender, age, and level of employment precariousness (see Table 2). Level of precariousness was included in the sampling strategy to ensure breadth in participant experiences and was based on established tools; the Employment Precarity Index (EPI) [52] and the Employment Precariousness Scale (EPRES) [53]. Additionally, each site aimed for variability in other criteria relevant to that context, such as household composition, ethnicity, educational level, and geographic location. More details are available, see Bosmans et al. [31].

**Table 2:** Demographic characteristics of interviewees (N =  250).

Country	Age	Lower level of employment precariousness (n = 124)		Higher level of employment precariousness (n = 126)	
**Female**	**Male/Gender variant**	**Female**	**Male/Gender variant**
Belgium	Younger (25-39)	5	6	6	5
Canada		6	4	6	5
Chile		4	5	5	4
Spain		5	6	5	5
Sweden		13	6	11	3
US		4	4	9	5
Belgium	Older (40-55)	7	2	3	4
Canada		5	3	5	6
Chile		5	6	6	5
Spain		5	5	5	5
Sweden		7	5	4	2
US		5	1	7	5

### Data collection

We conducted interviews between January 12 – September 9, 2021, using a semi-structured interview guide that queried several areas related to the workers’ experience of NSE, including links between those experiences and their health and well-being, strategies used to manage challenges, and policies and practices that supported their well-being (Supporting information S1 File). We conducted all but 16 interviews (in Spain) by video call. Interviews were conducted in appropriate languages for each region (Belgium – Dutch and French; Canada – English; Chile – Spanish; Spain – Spanish and Catalan; Sweden – Swedish and English; US – English and Spanish). Most interviews were 60-90 minutes; all were recorded and transcribed verbatim.

### Data analysis

We analysed data in two phases – (i) codebook thematic analysis of individual country data (single cases) guided by Braun and Clarke [54] in the language of data collection, and (ii) cross-case analysis of the six cases guided by Stake [50]. During the cross-case analysis process native speakers extracted and translated data from single case summaries into English (see Supporting information S2 File). For cross-country analyses, we used de-identified transcripts in accordance with obtained ethics approvals.

We used a combination of deductive and inductive codes based on prior research on NSE and its relationship with health and well-being. Codes related to employment experiences included employment stability, material rewards and psychosocial working conditions. Codes relating to health and well-being included self-reported physical and mental health as well as general well-being, the latter guided by the Centers for Disease Control and Prevention’s definition that encompasses positive and negative emotions about several aspects of one’s life [55]. Detailed information on the analytic strategy is available in Bosmans et al. [31].

As cases were compared and discussed, themes relating to labour and social protection policies were identified across all study countries. This led to the specific research questions addressed in this article, focusing on challenges identified by workers in NSE that could potentially be mitigated by labour and social protection policies, and workers’ responses to such challenges across the six countries. Codes relating to policy challenges included attitudes, barriers, knowledge and understanding of existing policies and suggestions for new policies. Codes relating to responses included economic and cognitive strategies. We assumed a realist epistemological approach to draw straightforward interpretations of participants’ experiences as shared in their spoken language [54]; our analysis thus reflects what was salient to participants. Although level of precariousness was considered in the sampling strategy, it did not emerge as central in relation to the research questions of this study during the cross-case analysis and is therefore not discussed further.

## Findings

### Gaps in protection

Despite the variability in country policies intended to support workers, many participants across all study countries described employment instability and income inadequacy, underscoring the importance of the link between NSE, material deprivation, and health and well-being. For example, most participants feared that their income could diminish significantly or disappear following a decrease in work hours, or the non-renewal or discontinuation of their contracts or work arrangements. A cleaning worker with fixed-term contract described this recurring concern (quotes have been edited lightly for clarity and include country code and interview ID, gender (M/F), and age):

*“When I stop working or when the end of the contract is approaching – you are already wondering if they will hire you in another place, the place you were before, if they will renew you […], and this creates constant anxiety over time, because if you have a contract of 6 months or less, as long as you have it, good, but as it ends, of course you’re already thinking that you will stop having a fixed income, and then it depends on what savings you have, because with 6 months you can’t save, and [you can’t save] with what you make either, it barely gets you to the end of the month.” [SP-19, F, 53]*

Similarly, participants across all countries pointed to employer practices that created or intensified uncertain employment conditions. For example, participants in most countries overwhelmingly referred to the uncertainty of both contract or work arrangement duration and work hours. A temporary university lecturer described how work hours can change on short notice:

*“We had one person come back from being off. My hours got cut, because she came back. Somebody else quit. My hours went up. Then we didn’t have an incoming class in January, because enrollment numbers were too low. So hours went down again. Everything really has an effect on me. And they’re [employer] not very well organized. They really do wait until the last minute to make these decisions. I’m usually the last person to know.” [CA-P07, F, 47]*

Some interviewees shared that employers avoid or reduce their costs by hiring mostly workers with temporary contracts, if relevant legislation exists it is rarely enforced, and employers find loopholes to circumvent it. Wages were often described as low or insufficient and income inadequacy in general was reported as limiting purchases directly tied to health and well-being including food, shelter, transportation, or clothing. This receptionist, also a primary caregiver to her sick brother, described how she struggles to make ends meet:

“*I don’t eat lunch, I’ll have oatmeal for breakfast. Then I’ll make a dinner that will feed my brother, myself, and I definitely prioritize my brother’s groceries over mine. Usually he gets the best stuff […] I’m selling basically whatever I can find around my apartment that my partner’s okay with letting go and just making sure that that’s turned into something that can add up to supporting either my brother and myself or my partner.” (CA-P23, F, 52)*

Some interviewees described abusive wage-related situations, and unauthorized immigrant participants mentioned even lower wage levels and wage theft (e.g., working longer than agreed upon for the same pay or not being paid) that they endured given their unprotected status. Similarly, some interviewees working through internet-based platforms and temporary work agencies reported high administrative fees that reduce their pay, and some self-employed workers shared instances of delayed client payments.

Inadequate or insecure housing was an often-cited problem that participants across all countries directly linked to the employment instability and income inadequacy rooted in NSE. For instance, many described not only unaffordable rents but also how being in NSE limited their access to credit to purchase a home or secure a rental contract. In addition to barriers due to inadequate or unstable earnings, interviewees in several countries expressed that qualifying for a mortgage or a rental contract is dependent on having a permanent work contract or reliable employment sources, for example this self-employed artist from Sweden:

*“You almost need to lie about your [employment] situation to get a place to live even though you know that you work hard and are well behaved and do everything right. It feels like… if you are interviewed by a culture magazine it would say ‘you are doing so well’ but if you ask a landlord, you are not worth anything.” [SE-29, F, 33]*

In addition to health stressors arising from inadequate or insecure income, other frequently mentioned gaps were related to a lack of or limited employment-related rights and protections specifically tied to health. Whereas country-level data showed differences in workers’ rights to paid or unpaid vacation, parental and sick leave, interviews suggested that many participants in all study countries refrained from exercising certain employment-related rights, even when available by law, due to fear of negative job repercussions, as illustrated in this teacher’s experience:

*“At work I cannot say I am sick. When I have an episode and strong headache, I must endure it, because it’s like, “Ooh, you’re a burden” [implying employer’s response]. That’s my biggest concern, I think. If something serious happens to me, where do I get money to live while I can’t work?” [CL-12, F, 34]*

Accessing paid leave was also often cited as problematic due to regulatory restrictions that rendered workers ineligible or made it difficult to use such benefits even when the right existed on paper. Some interviewees were allowed to take parental leave, for instance, but pay was minimal due to lack of full-time work hours and corresponding qualifying income. The need to work for the same employer for a pre-determined time similarly barred others from this benefit. Complicated rules and lack of information were also described as barriers. A restaurant worker and recent US immigrant shared her frustration with her managers and employer’s leave policy in which sick days and paid time off are combined:

*“The fact that you go to ask several times and they [managers] do not know the information, I don’t have the benefit of sick time that I would like to take, I do not know if it is okay, if the system is like that… I know very little about the labour schemes here [in the US].” [US-49, F, 43]*

Moreover, not only did most study participants experience less job stability, but they also reported that their right to unemployment support between jobs was irregular. While Belgium, Spain, and Canada (in that order) offer higher levels of unemployment income support to workers when compared to the other three countries, most non-self-employed participants described either not having access to comparable amounts of unemployment benefits as standard workers or not having access at all. Self-employed participants in all six countries reported lacking unemployment benefits, sometimes despite paying into social security programs:

*“Your safety net is actually non-existent. There’s no unemployment benefit. Everyone just always assumes that if you’re self-employed, that you’ll make a lot of money... And yes, there are times when you do well and times when you don’t do so well [...] the only thing you do is pay a lot of social security contributions and, I actually pay as much as an employee, but there is actually nothing or very little in return.” [BE-2, F, 50]*

Another illustration of limited rights and protection is how most participants across all countries expressed concerns about weak union protection. This similar pattern seemingly contradicts the country-level data indicating relatively large differences, with high union coverage in Belgium and Sweden, and the US and Chile lagging far behind. The interviews revealed that many unionized and non-unionized participants did not perceive unions as helpful in improving their employment conditions. This temporary agency worker described why unions would not be helpful in his situation:

*“Everyone is replaceable and that is a big advantage for the employer. You can be the best worker there is, but if I say in a company: “Tomorrow I’ll go to the union because that and that is totally wrong”, then they’ll [the employer] say: “Go to your union”. They don’t care about that at all. Your contract ends on Friday, and on Monday you don’t have a new contract as a temporary agency worker. On Monday, there will be someone else standing there who wants to come and work, and he doesn’t know how bad things are, or maybe it’s a totally different person who won’t open his mouth.” [BE-5, M, 32]*

Some interviewees referred to unions as unavailable or banned. Specific concerns varied and included complaints about unions mostly representing the interests of standard employees and being less relevant for workers in NSE. Other union-related concerns were, for instance, failure to enforce existing rules (e.g., overtime pay) and workers being impeded from joining unions due to non-standard contracts. The most egregious barrier was shared by a few workers who expressed that participating in a union was strongly discouraged, potentially threatening their jobs.

Despite many similarities we found some notable differences, especially within policy domains where some countries adopted a more universal and non-employment-based approach compared to others. Specifically, interviewees in the Americas described unequal access to health care and issues with childcare, both fundamental social protections that European countries in our study provide, universally. Country-level data indicated that working populations in all study countries, save for the US, have relatively high levels of access to health care, yet most of our interviewees in Canada and the US mentioned that health coverage is costly and out of reach, since some or all health coverage, in the case of US, is employer-based and predominantly reserved for those in full-time, standard work arrangements. Additionally, while not a consequence of NSE, participants with family care responsibilities in these same countries attributed their need to pursue flexible NSE to a lack of universal or affordable childcare and eldercare services.

### Workers’ responses to gaps in protection

Participants across all study countries responded to policy gaps in strikingly similar ways by relying on their individual resourcefulness, for example by using individual and family resources. Since financial unpredictability was a great concern, most interviewees considered personal savings to be an important buffer in times of job loss, inability to work due to sickness, or decreased work hours. Overwhelmingly, workers in all countries described how family resources were an important source of support, such as providing access to health coverage, and other financial buffers including childcare and housing; some participants lived with family or in their property. The importance of this support is emphasized by this seasonal worker:

*“Well, if I didn’t have my family, I would be living on the streets or stealing or committing crimes or any of these things, I’ve told you that, my family is my salvation [...] Here we help each other, that is, if you don’t have, I have, if I don’t have, you have.” [SP-5, M, 55]*

Additionally, participants across all countries engaged in multiple strategies to counter employment instability and its consequences. Workers described spending considerable unpaid time procuring work, accepting worse working conditions such as high-volume workloads, tight deadlines, and lifting heavy equipment, or working more hours than specified in their agreements, without overtime pay. Moreover, even when the law provided access to paid or unpaid leave, participants mentioned avoiding taking advantage of it for fear of job task change, loss of hours, or complete job loss. Such strategies were often commingled with purposeful management of relationships with managers or clients to avoid hurting their chances of getting work:

*“I don’t want to have any disagreements with [my supervisor], I just want to fly under the radar. Because you are replaceable. It’s not worth it. […] The few times she comes to the packing site, you just put on a big smile. You don’t exactly suck up to her, but you make sure to be very nice, because you know it gives you better shifts.” [SE-10, F, 27]*

### Workers’ vision for policy changes

We asked participants to envision potential supportive changes in policies or practices. While some workers had clear visions for improvements, most tended to struggle to raise ideas on their own and had relatively few suggestions given the multi-faceted challenges they experienced and the considerable effort they expended to address them. This interviewee, an artist combining different part-time jobs, found it difficult to envision solutions while dealing with the fundamental worry she shares with others:

*“It’s difficult to think about that now [suggested improvements] because all energy is consumed by just making ends meet and getting enough money to get through the month.” [SE-20, F, 30].*

Nevertheless, when prompted on the topic, participants shared several ideas. Some pointed to a general need for stronger regulations to counter employment and income instability, for example through more stable, predictable, longer-term contracts or arrangements.

*“I believe that we need to generate more stability, because, unfortunately, as we are under contract for services, we do not have stability or we do not have a contract to say: “I earn, for example, this monthly amount and I work in such and such institution.” [CL-22, M, 31]*

Participants in several countries suggested the need to regulate a minimum or maximum number of working hours to counter income inadequacy or, at times, overwork. Some participants highlighted employers’ responsibility to mitigate the effects of NSE and called for more transparency and foresight regarding contracts and scheduling, and better employer-based policies (e.g., access to rest spaces, pay transparency, and benefits such as paid sick time, or paid time off to care for ill family members). Some also suggested specific measures aimed at employers, for instance, holding employers accountable to minimize misuse of temporary contracts.

Another idea discussed across most countries was the introduction of universal basic income, based on participants’ beliefs that it would reduce stress from employment instability and income inadequacy, though not everyone thought it would be tenable. A participant who worked as a theater actor but also had multiple other jobs encapsulated this sentiment:

*“It would be helpful to everyone. It would be helpful to people that need money. It would be helpful to people, unemployed and looking for work. It would be helpful to people living on the streets. It would, of course, be helpful to everyone. I just don’t, unfortunately, see it happening.” [US-23, M, 28]*

Improvements in health policies were rarely mentioned by participants in the European countries, where health coverage is universal. In contrast, concern about affordable health care prevailed for participants in Canada, the US, and in Chile where especially insufficient coverage of mental health care was described. US workers desired access to affordable health insurance in general, whereas Canadian participants wanted coverage beyond basic universally provided hospitalization services (e.g., prescription medication and vision services that are provided as employer-based benefits).

While participants across study countries expressed general support for family care benefits and the potential role of labour unions in protecting workers’ rights and benefits, very few participants discussed concrete policy improvements for those specific domains. Furthermore, participants across all countries generally expressed their lack of faith that anything would change.

Upon closer analysis of the participants’ responses across contexts, their narratives either reflected a rights-oriented or a normalized perspective. For instance, interviewees from Belgium, Chile, Canada, Spain, and Sweden expressed their rights to protections, referring to their situations as unjust or needing to change, while often begrudgingly accepting them. Workers with this rights-oriented view highlighted ‘gaps in the system’ and suggested that policies were designed for standard employees, and poorly adapted to the realities of workers in NSE. A self-employed IT-consultant shared her frustration:

*“I still pay as much tax as anybody else, so that tax money should be there to help me, but since I have chosen not to settle in this ‘hamster wheel’ [standard employment] I just have to accept that I don’t get access to the safety net. And I have accepted it, even if I think it is wrong.” [SE-21, F, 37]*

In contrast to this rights-oriented view, workers from the US, where workers have few guaranteed labour rights protections [56], tended to discuss their challenges with tones of acceptance, seemingly normalizing the lack of support. This contrast suggests that the country contexts may shape what a worker will expect from the state, unions, or employers. US interviewees tended to seek employers that could potentially offer better alternatives rather than naming legislation or governmental responsibility. A part-time technical designer for a clothing company explained multiple trade-offs she was planning to accept to obtain time off:

*“I want more vacation time, instead of more pay. Or I want one day off during the week […] I’m going to accept it; it’s going to be fine. I won’t even say anything […] If my job is secure, I’ll work like that […] My company’s been good to me, especially compared to other companies.” [US-123, F, 41]*

## Discussion

### Key findings

In this study, we explored the experiences of workers in NSE in six high income countries across three continents, with diverse levels of labour market regulation and social expenditures. Despite several substantial differences in existing labour and social protection policies, workers across the six countries reported experiencing overarchingly similar policy gaps with negative implications for their health and well-being, including employment instability, income inadequacy, and limited employment-related rights and protections. Given these gaps, workers across all countries tended to respond in similar ways, relying on individual resources and strategies rather than on government or employer support, an approach which can further compromise their own and their family’s health and well-being. Workers’ experiences in NSE point to clear policy gaps, even in countries with strong labour legislation and generous social safety net programs, such that workers are either denied or forced to accept inferior protections to attain some level of employment stability. Despite clear gaps, workers made relatively few suggestions for concrete supportive policies or practices and expressed low expectations that policy changes were likely to occur.

### Similar experiences across policy contexts

Our policy context overview examining labour and social protection policies across the six countries highlight clear differences that are consistent with the economic typologies identified in the varieties of capitalism approach [45] and welfare regimes literature [42–44]. Based on these comparisons, we expected to find more variability in participants’ experiences with employment-related protections, but surprisingly, workers highlight interesting similarities. The similarities in experiences across countries suggest that employment-based policies are not tailored in a way that benefits workers in NSE in practice [34,35]. Our findings highlight that although study countries have a spectrum of labour and social protection policies, these have been modelled predominantly for standard employment arrangements and have not been adapted or are not adequately enforced to meet the needs of workers in NSE. Thus, while, in theory, these protections exist for workers in general, in practice they do not exist for many workers in NSE, or even when they do, workers encounter barriers to benefiting from them. This illustrates a point previously made by Roberts and colleagues (2015); that drawing exclusively on country-level data in policymaking may misrepresent reality and disadvantage certain subpopulations whose needs and vulnerabilities remain unnoticed [57].

A common theme across all countries was the perceived power imbalance preventing participants from claiming employment-related rights. As a result, many participants did not benefit from existing labour regulations and social protections even in countries like Sweden, that according to our policy context overview ranked highly on protective legislation. This finding suggests that while there is certainly a lot that can and should be done to strengthen workers’ rights and improve employment-based social protection (although the details of such policies are out of scope for this paper), stricter regulations will be difficult to enforce and should not be the only focus for solutions.

By contrast, our analysis showed that universal policies were more successful at benefitting workers in NSE. Universal health care is a good example as participants in Europe rarely mentioned any challenges related to this topic whereas other participants, most notably in the US, described health care related costs as a great cause of stress. This finding underscores the benefit of providing basic social protection outside of the employment contract. It also illustrates another broad pattern found in our data that we explore in another publication: while we found striking similarities in workers’ experiences of policy gaps and relative disadvantage compared to those in standard employment within the same country, participants in countries with more generous welfare states generally navigated the challenges with greater ease [31].

### Workers’ responses and vision marked by their need to fend for themselves

In the face of employment instability and economic fragility, participants across all countries needed to expend considerable individual effort protecting their health and well-being by managing unstable labour and living conditions. Their surprisingly few ideas for policy improvements may be a symptom of this mental effort standing in the way of even imagining potential policy or regulatory changes. The similar pattern can be explained by all study countries being characterized by the same neo-liberal tendencies accelerating the “precaritization” in the labour market [1,2], including a shift of economic risk from employers to workers as we found in our study. In reflecting on this phenomenon in the US context, Hacker (2019) suggests that individuals and families have increasingly borne the risk of responding to fluctuations of the market-based economy, forcing them to fend for themselves in times of job loss and associated loss of employer-based social protections [58]. Similarly, in the European context, Standing (2021) argues that neoliberal policies are the main driver behind the emergence of a ‘Precariat’, who are workers with eroded rights, lack access to benefits, and whose lack of employment security can result in, for example, unsustainable debt, as employers shift the financial risk of downturns in the business cycle from themselves onto the workers [59]. To compensate, precarious workers, such as many in our study, need to spend considerable time in pursuit of work opportunities, i.e., “work for labour” [59, p.141]. Moreover, participants’ difficulty envisioning change and low expectations can also be interpreted as a symptom of perceived distance from political power, adding another barrier to taking action. Standing argues that precarious workers have not succeeded in mobilizing change due to a lack of political identity and representation [59]. In line with this our findings show that historically important institutions of worker power, such as labour unions, were perceived by most participants to lack actual power, including by participants from countries with high union density such as Sweden and by those employed in unionized workplaces.

### Differing perspectives: workers’ rights vs. normalization

While our findings showed many similarities across the six country contexts, we found a notable attitudinal difference among participants when prompted to envision supportive policy changes. Workers from five countries tended to use language that reflected their belief that all workers should be guaranteed basic labour rights from which they often felt excluded, while workers in the US were more likely to accept their relatively low level of rights as a normal part of employment. In comparison to the other study countries, the US fares least favorably on most labour protection indicators. Despite low expectations for change, participants in Europe, where more protection is provided to workers in general, pointed to employment-related problems and could more easily define potential improvements, such as laws that would remove barriers and create better enforcement of existing labour rights. This contrast suggests that the country contexts may shape what a worker will expect from the state, unions, or employers. In light of this, it is not surprising that US participants focused more on individual solutions to manage their situations compared with the European participants.

### Non-Standard Employment in the context of COVID-19

Recent evidence suggests that the economic effects of the COVID-19 pandemic worsened material conditions, health, and well-being for many workers in NSE and in other forms of precarious employment [25,60,61]. While it is beyond the scope of this paper to focus on the influence of COVID-19 policies, country-level COVID-19 measures may in some ways have flipped the table, creating a situation in which participants in countries with less generous policies got access to several sources of financial support they had not benefitted from before [62], while participants in countries with traditionally generous welfare policy contexts realized for the first time how inaccessible the existing support systems could be for them. In both cases, COVID-19 worked as a stressor exhibiting the weaknesses of regulatory frameworks and social protection that in strong welfare contexts may otherwise have gone unnoticed, and that in weak welfare contexts prompted a response workers would not have imagined in a normal situation [63]. In this sense, while collecting data during the pandemic may make it difficult to disentangle COVID-19 from the regular NSE work situation in the narratives of our interviewees, it may have placed us at the point of a global historical shift in the perceptions of workers concerning the levels of employment and social protection to which they are or could be entitled. In the coming years in-depth studies of whether this is a lasting effect of the pandemic response are needed.

### Implications for improving population health and well-being

Workers in NSE require similar forms of protection against economic risks and violations of workers’ rights as those in standard employment, but our study highlighted significant policy gaps with implications for workers’ health and well-being. Our findings also indicated that when other supportive policies, e.g., health care and childcare, are universal and not contingent on income or employment, workers benefit, and the consequences of lacking individual and family resources are less severe. Therefore, while acknowledging that efforts need to be multifaceted addressing social programs, material rewards, working conditions etc., our findings indicate that a supportive policy approach would be to shift the provision of key forms of social protections and welfare guarantees out of employment contracts and move towards more universal forms of social protection [38,64]. Such an approach would especially benefit those with lesser market power or in more fragile labour market positions, and hence less capacity to fend for themselves.

Further, the identification and implementation of successful and sustainable solutions to improve population health and well-being require not only the recognition that employment is a structural social determinant of health [65,66], but the acknowledgment that employment quality and employment relations are influenced by power dynamics within socioeconomic and political contexts [67].

Finally, it is important to underscore the need to explore the consequences of NSE beyond the pecuniary functions of work, and explore its latent functions, such as meaningful relations at work [68]. Such attempts could allow us to fully understand the psychosocial and health consequences of employment flexibility, precariousness and contractualization, and to advance our knowledge in order to promote work-related well-being.

### Limitations

Our recruitment strategy, which focused predominantly on use of social media, may have excluded some workers in NSE from participating in the study, including workers who do not have access to social media or speak the languages used in the study. On the other hand, our strategy allowed us to reach a wider audience of workers than would have been possible through more traditional outreach methods, especially given the dispersed and informal nature of many of the participants’ employment arrangements. Nevertheless, further research should target these harder to reach groups of workers to gain more insights on their potentially differing experiences.

We acknowledge that the quality of interview data can vary depending on the method of data collection. Video-call interviews allow for both verbal and non-verbal communication cues and thus provides rich data, but technical glitches may also interrupt the natural flow of the conversation and cause frustration, especially if a participant is less familiar with such technology. Our guiding principle was to prioritize the comfort and convenience of the interviewees, and, accordingly, all participants were offered the choice between video-call and face-to-face format. Sixteen interviewees in Spain chose face-to-face format. Although we cannot rule out that this variation in data collection format may have impacted the findings, we believe that allowing participants to choose the interview format contributed to their comfort and rapport-building and thereby to the richness of the data.

Our comparison of country policies is based on data reported to international agencies but may not reflect the reality of what is implemented, enforced, or experienced, even by more privileged workers in standard work arrangements. The qualitative data counterbalances this limitation by revealing the participants’ own perspective and experiences.

The recruitment was targeted to certain parts of the countries and the findings may not represent variations within each country due to regional laws and policies. However, the aim of this study was to compare policy contexts and given the large differences between the study countries, intra-country regional variation is unlikely to bias the findings [69].

The purpose of this study was to provide an overview picture of workers’ lived experience with gaps in policy and practice, and the data are not suited for any detailed policy analysis. Future studies should delve deeper into the impact of specific types of national or regional policies on respondent trajectories and on their health and well-being.

Finally, while asking workers for their thoughts about supportive policies was fruitful, our methodological approach may not have been sufficient to capture the depth of their ideas. The questioning process for phenomena that is not usually part of people’s day-to-day lives could be enhanced by utilizing participatory visioning exercises [70]. Notwithstanding, the differences in participants’ attitudes to protections and rights that emerged in our study may point to future intervention research that explores how policy changes could be approached in each country context.

## Conclusions

As economic globalization and competition drive employers to restructure jobs, forcing many workers into more temporary and flexible work arrangements, protective labour and social policies need to adapt to ongoing changes in the way work is organized and structured. However, our study identified significant policy gaps with implications for workers’ health and well-being across high income countries irrespective of the levels of labour market regulations and social protections. Furthermore, the shifting of responsibility for financial and social support from government programs to individuals and their families, as described by participants, is likely to exacerbate health inequities, especially for those with fewer resources and lower social position. This study highlights challenges with existing policies such as access barriers and difficulty enforcing rights due to power imbalances. To promote health equity, efforts to improve social protection for this vulnerable population should be prioritized.

## Supporting information

S1 Table Comparison of labour market regulations and social protection indicators - detailed scores, measurements, and data sources.
Detailed version of the Table 1
(DOCX)

S1 File Semi-structured interview guide.
The interview guide in English
(DOCX)

S1 Checklist Inclusivity in global research questionnaire.
(PDF)

S2 File Analytical strategy of multi-country individual worker data.
Detailed description of the qualitative analysis
(PDF)
