# Supplementary material for: Labour and social protection gaps impacting the health and well-being of workers in non-standard employment: An international comparative study
Source: PLoS One. 2025 Mar 25;20(3):e0320248. doi: 10.1371/journal.pone.0320248 (PMC11936240; doi:10.1371/journal.pone.0320248)
Supplement: S1 Table — Detailed version of the Table 1 (DOCX) [file pone.0320248.s001.docx]

**Manuscript title:** Labour and social protection gaps impacting the health and well-being of workers in non-standard employment: An international comparative study

**Supportive material S1 Table. Comparison of labour market regulations and social protection indicators - detailed scores, measurements, and data sources**

| Indicator | Canada | US | Chile | Sweden | Belgium | Spain | Year | Measurements |
| --- | --- | --- | --- | --- | --- | --- | --- | --- |
| **Labour market regulation indicators** | | | | | | | | |
| Labour market regulation Index*^1^ | 4.02 | 3.29 | 9.35 | 8.54 | 9.46 | 11.07 | 2019 | Scale 0-30. Sum of five indicators listed below |
| Components of the labour market regulation index | | | | | | | | |
| Strictness of employment protection of regular contracts^1^ | 1.61 | 1.30 | 2.45 | 2.48 | 2.72 | 2.43 | 2019 | Scale 0-6. Individual dismissal protection. Average of 12 indicators. |
| Strictness of employment protection of temporary agency and fixed term contracts^1^ | 0.28 | 0.33 | 2.42 | 1.67 | 2.17 | 3.10 | 2019 | Scale 0-6. Average of eight indicators. |
| Regulation of collective dismissals of regular contracts^1^ | 1.85 | 1.33 | 2.06 | 2.72 | 2.40 | 2.43 | 2019 | Scale 0-6 |
| Regulations for hiring temporary agency contracts^1^ | 0.28 | 0.33 | 1.42 | 1.29 | 1.79 | 1.73 | 2019 | Scale 0-6 |
| Regulations for hiring fixed term contracts^1^ | 0 | 0 | 1.00 | 0.38 | 0.38 | 1.38 | 2019 | Scale 0-6 |
| Other labour market indicators | | | | | | | | |
| Active labour market expenditure^2^ | 0.22 | 0.10 | 0.14 | 1.25 | 0.88 | 0.68 | 2017 | % of GDP. This includes retraining |
| Passive labour market expenditure^2^ | 0.56 | 0.14 | 0.37 | 0.53 | 1.39 | 1.51 | 2017 | % of GDP. This includes unemployment insurance |
| Jobless income benefits^2^ | 21.0 | 6.0 | 4.0 | 47.0 | 40.0 | 28.0 | 2017 | % of median disposable income for a single person |
| Paid sicks days index** ^3^ | 77 | 27 | 102 | 120 | 114 | 105 | 2020 | Summary index. Average of 25 indicators. |
| Minimum wage ^2^ | 21,280 | 15,080 | 7,330 | n/a | 22,872 | 17,891 | 2019 | $US ppp |
| Union density: private sector^4^ | 14.8 | 6.5 | 12.9 | 64 | 53 | 14 | 2013-17 | % |
| Union density: workers with temporary contracts^4^ | 26.9 | 6.0 | 6.6 | 39.3 | 49.2 | 4.6 | 2013-17 | % |
| **Social protection indicators** | | | | | | | | |
| Overall social expenditure^5^ | 17.4 | 18.2 | 10.9 | 26.1 | 28.9 | 23.7 | 2018 | % of GDP. (Cash benefits, direct in-kind provision of goods and services, and tax breaks with social purposes) |
| Specific indicators of social protection | | | | | | | | |
| Income support to working age population^5^ | 4.6 | 1.9 | 1.8 | 4.0 | 7.5 | 4.9 | 2015-17 | % of GDP. (Incapacity benefits, family cash benefits, unemployment, and other social services) |
| Population coverage for health care^5^ | 100 | 35.9 | 75.6 | 100 | 98.7 | 99.0 | 2017 | Total public coverage % |
| Paid maternity, parental and home care leave available to mothers, *duration* (weeks)^5^ | 51 | 0.0 | 30.0 | 55.7 | 32.3 | 16.0 | 2018 | Number of paid weeks |
| Paid maternity, parental and home care leave available to mothers, *amount* (average payment rate)^5^ | 52.1 | 0.0 | 100.0 | 62.1 | 40.4 | 100.0 | 2018 | % (level of income replacement) |
| Pension spending (public)^5^ | 4.70 | 7.06 | 2.82 | 7.17 | 10.71 | 10.02 | 2017 | % of GDP |

Colour codes relative to the average for 6 countries:

More than 25% above six-country average

Between 10-25% more than six-country average

Within plus minus 10 % of six-country average

Between 10-25% below six-country average

More than 25% below six-country average

*We calculated the Labour Market Regulation Index as a sum of five indicators, included separately in this table: (1) strictness of employment protection of regular contracts, (2) strictness of fixed term and agency contracts, (3) regulation of collective dismissals of regular contracts, and (4 & 5) regulations for hiring temporary and fixed term contracts. The individual value for each of the five indicators for each country is displayed as a score from 0 (weak) to 6 (strong) and was extracted as is from the 2019 OECD data. Two of the indicators used to calculate the Labour Market Regulation Index are an average of other indicators. ** We calculated the Paid Sick Days Index as an average of 25 indicators, provided by the WORLD Policy Analysis Center at UCLA as ordinal rankings (1=low & 5 = high) for multiple features of the paid sick leave policy.

Indicator sources:

^1^ OECD Employment and Labour Market Statistics database, 2019 data (<https://www.oecd-ilibrary.org/employment/data/oecd-employment-and-labour-market-statistics_lfs-data-en> ).

^2^ OECD Employment and Labour Market Statistics database 2019 (<https://www.oecd-ilibrary.org/employment/data/oecd-employment-and-labour-market-statistics_lfs-data-en>).

^3^ WORLD Policy Analysis Center UCLA, database, Short Term Sick and Medical Leave 2020 data (<https://www.worldpolicycenter.org/maps-data/data-download/short-term-paid-sick-leave-data>).

^4^ Union density: J. Visser, ICTWSS Database. version 6.0. Amsterdam: Amsterdam Institute for Advanced Labour Studies (AIAS), University of Amsterdam. June 2019. (<https://www.ictwss.org/downloads>).

^5^ Social protection indicators: OECD Social Spending data base (<https://data.oecd.org/socialexp/social-spending.htm>; <https://www.oecd.org/social/soc/OECD2019-Social-Expenditure-Update.pdf> ). Based on the most recent data available (2015-2019).
