# Supplementary material for: Labour and social protection gaps impacting the health and well-being of workers in non-standard employment: An international comparative study
Source: PLoS One. 2025 Mar 25;20(3):e0320248. doi: 10.1371/journal.pone.0320248 (PMC11936240; doi:10.1371/journal.pone.0320248)
Supplement: S1 File — The interview guide in English (DOCX) [file pone.0320248.s002.docx]

**Manuscript title:** Labour and social protection gaps impacting the health and well-being of workers in non-standard employment: An international comparative study

**Supporting information S1 File. Semi-structured interview guide**

The interview guide below was developed in English. Each country team translated it as needed and adapted terminology and prompts to be appropriate for their country. The guide queried following areas: (1) the experience of the employment situation, (2) the reasons for undertaking NSE, (3) perceptions of links between their employment situation and their and their families' health and well-being, (4) links between the employment situation and the COVID-19 epidemic, (5) the strategies used to cope with difficulties, and (6) policies or practices that support their and their families' health and well-being.

For each of these, we formulated a general question that was used in every interview. Specific prompts related to each general question could be used by the interviewers as they saw fit. This was a choice made to balance a need for similarity of inquiry across cases and for tailoring to the specificities of each participant. Training sessions were held so that each country team could understand the guidelines for questioning. Inspired by phenomenological approaches [1], we paid special attention to the interviewees' meanings, feelings, and opinions, and how they made sense of their situation to capture the experience of non-standard employment.

**Interview guide**

# **Material to bring or prepare**

- Interview guide: Canada and Sweden re-formatted extensively to make it user-friendly, each site can re-format as they see fit.
- Informed consent forms
- Pen
- Blank sheet
- Recording: follow ethics and/ or institutional requirements for video conferencing software and appropriate storage of files. To ensure good quality audio, place the computer near wireless modem/ router, or use a wired connection.
- Use an audio recorder (+ batteries) for back-up recordings.
- Optional if needed to prepare and depending on interview mode in each country: money/gift voucher, chocolates, masks, etc.

# **Introduction to interview**

- Thank the respondent for coming
- Introduce yourself (interviewer, researcher) briefly (your role in the project)
- Explain aims of the project: Research into how non-standard employment is experienced during the COVID-19 crisis, how it influences health and wellbeing of the non-standard employees and their families, what policies or practices could support the health and wellbeing of non-standard employees, and what motivates workers to undertake non-standard employment. Compare results of interviews in 6 different countries (Sweden, Belgium, Spain, USA, Canada and Chile).
- Go over the information form and manage consent according to your country’s approvals.
- Explain reason for recording audio (don’t want to miss any thoughts or opinions, time to take notes to see what we have already discussed, ability to listen to interview later, info from interview and quotes will be used anonymously in publications) + obtain permission. If relevant, explain that video will not be maintained, only audio.
- Inform that the interview will last approximately 1 to 2 hours
- Inform that the interview is an open conversation, that you will guide discussion on the basis of a number of questions and in which the interviewee can say everything that comes to mind. Let the interviewer know that at times you may ask for clarification or elaboration. This is to be sure that you have clearly understood what the interviewee means to say, or to gain more information about something particularly important.
- Put respondents at ease by saying that there are no right or wrong answers. Explain that the most important thing is what they think, feel, experience, how they deal with what they experience, etc.
- Any questions?
- Inform that you will start the audio recording

## Preliminary data

Note interview country; review of survey data prior to interviews depends on ethics approval at each site. If you, the interviewer, cannot access these data, follow a pre-determined process to communicate about the participant with other team members.

Ask to introduce them

- What they would like to be called in interview (first name or some other choice)
- Gender
- Age
- Education
- Ethnicity
- Professional/employment situation
- Household composition (+ gender, age, education, ethnicity, professional/employment situation of other household members)

# **Interview guide**

*Interviewer guidelines:*

- *The* ***main*** *questions in this interview guide should be all asked and about the same way in each interview for comparability purposes.*
- *Prompts and form of address are open to appropriateness of the society and the individual interviewee.*
- *Prompts should be handled carefully. The prompts and lists of examples should not be presented as a list of choices which people must address. Prompts can be used either to help the interviewees get going (if they do not answer the questions in an 'open way'), or to probe further.*
- *This guide is meant to assist you in the questioning process and does not have to be followed in the order as it is written if the participant begins to talk about topics that are listed later in the interview guide; let them talk as they see fit. You can return to those topics to discuss them more profoundly afterwards using the questions and prompts further below. An example of this is when the coping question is asked. You can follow the participant’s lead whenever they raise this, ask the question as in the order it is written, or towards the end of the interview.*
- *If participant becomes emotional at any point in the interview, offer words of acknowledgement about difficult circumstances as appropriate to local culture and language., e.g. “it is a difficult situation,” “it must have been hard.” Ask them if they would like to take a break. And, keep handy a prepared a list of different kinds of service/ organizations for referrals. Offer them the name and contact information of relevant organization(s) during the interview or at the end depending on the flow. You can share in the chat box, via email, or this may have already been included in the consent document that you can reference again.*

**Experience of employment situation**

From the questionnaire you filled out and our preliminary conversation here, I know a little about your employment situation. Could you tell me now about what this employment situation is usually like for you in the day-to-day?

*Interviewer guidelines:*

- *Keep participant focused on aspects of employment arrangements and conditions, and less on working conditions and work content.*
- C*hoose appropriate prompts based upon whether the person is self-employed or an employee.*
- *People can have multiple jobs or non-standard employment positions. If this is the case, try to talk about the different jobs/positions and how they are combined. Make sure you always clarify which job/position the participant is talking about in any given comment.*
- *If the participant has changed jobs (different employment situation compared to the situation in the screening survey) and is not in non-standard employment anymore, ask the questions referring to the previous situation (non-standard employment). Additionally, ask how and why they changed their employment situation.*

Prompts:

In particular, I am interested in your experiences of things like:

- The stability of your employment situation (e.g., kind of contract, employment stability)
- Your income? Benefits? The stability/predictability of your income?
- Your rights and social protections (social security, legal protections)?
- Your working hours?
- Your control or influence over working hours, other conditions at work?
- Your training/development opportunities?
- The relationship between you and your employer? [for employees]
- The relationship between you and your colleagues?
- The relationship between you and your clients or other subcontractors? [for self-employed/entrepreneurs]
- Opportunities for representation at work? (e.g. workers’ collectives, professional interest groups, trade union)

*Clarify/probe about aspects of the employment situation specific to the COVID-19 epidemic and economic crisis*

We are interested in understanding the impact of the coronavirus outbreak and its aftermath on

employment. Which of the things you just described would you say were changed by COVID-19?

*Interviewer guidelines:*

- *If it feels more natural to clarify as the participant is describing their current employment situation, and if they were in the same arrangement before the pandemic as they are during the interview, you can ask them if what they are describing was their experience before COVID-19. But the question below should also be asked.*

In what [other] ways, if any, has your employment situation been affected by the turbulence following the worldwide spread of coronavirus?

*Interviewer guidelines:*

- *Because of the different ways countries have responded to COVID-19 (e.g. taking national action, leaving it to smaller units of the country, or letting employers make their own choices), for anything the participant brings up, be sure to clarify whether this is an employer-initiated action or one coming from some level of government.*
- *If possible/respondent knows, clarify whether it is a state/province/autonomous community/regional/lands action or a national one, as relevant to your country.*

Prompts:

- Things that might come to mind could be
- Layoffs
- Impact on income
- Working hours, mode of working
- Lack of economic, health, or social protection (e.g. remedies for lost income, ability to stay home if ill or potentially exposed and still be paid, if family member is ill)
- Access to healthcare
- Lack of physically protective conditions for the job (e.g. gloves, adequate personal space)
- Lack of information
- Barriers to comply with information/guidelines
- Worries, stress
- Impact on family (e.g. work-life balance, childcare, home schooling, domestic violence)
- Impact on housing
- Impact on food security
- Isolation and social support

*Reasons for undertaking non-standard employment*

How did you get into this kind of employment situation?

*Interviewer guidelines:*

- *If the participant responds with health and wellbeing concerns, guide them to return to descriptions about work (or unemployment) that brought them to their decision: “You talked about [health/wellbeing issues], can you tell me about the specific circumstances that influenced your decision to do the work you currently do?”*

Prompts:

- What were you doing at the time you started this employment, or before that?
- I’m thinking about what your circumstances were at the time you started this employment, what factors you considered? Were family obligations part of it? Did it have anything to do with the pandemic [if relevant]?
- What were you hoping to do/achieve/gain with this employment? (e.g., how did it fit into building your career over time?)
- Is this the type of employment you prefer? Would you prefer a standard job? If so, were you ever turned away from a standard job? Can you explain why?

**Perceptions of links between employment and health/wellbeing for workers and their families**

In what ways, if any, might your employment situation be related to your health and your family’s health in the day-to-day?

*Interviewer guidelines:*

- *Follow the lead of the participant – use worker-specific or family-specific prompts based on what participant focuses on.*
- *If participant* ***does not*** ***mention any difficulties****, use a prompt such as this, “You have told me about some ways that your work makes you feel [use their words as much as possible about what they said that was neutral or positive].  Are there any times you can think of where your work may have impacted you less positively?" Do not probe further if the participant still does not mention difficulties.*
- *If participant mentions coping/managing, ask about them as they mention them or later when encountered on the guide.*
- *If participant* ***only mentions difficulties****, ask the prompt above in the opposite manner. For instance, “You have told me about some ways that your work makes you feel [use their words as much as possible about what they said that was negative].  Are there any times you can think of where your work may have impacted you positively?"*

Prompts:

- If relevant: How well you have felt over time being in this employment situation?
- Examples of worker health and wellbeing issues for prompts if needed:
- Feelings of freedom, personal control, independence
- Flexibility to manage other aspects of life
- Job satisfaction
- Physical health (e.g. musculoskeletal complaints)
- Psychosomatic complaints (e.g. headache, stomach pain, palpitations, abdominal cramps, painful neck muscles)
- Biorhythm disorder, sleep problems
- Mental health
  - Mental wellbeing
  - Feeling anxious or on edge, depression
  - Stress
  - Burnout
  - Addictions (e.g. alcohol, drugs)
- Occupational diseases
- Injuries at work
- How has your employment situation affected the relationship with your family members?
- Examples of family health and wellbeing issues for prompts if needed:
- Feel like they come second to work
- Feel as if they are distracted by your employment circumstances
- Their sleep/meals/social life is disrupted by your schedule
- Their moods are affected by your moods/strain
- Your intimate life with a partner is impacted
- Your children are impacted
- An impact on the amount of care you are expected to give others (care work)
- Greater difficulty reconciling paid work and unpaid work

*Clarify/probe about links between employment and health specific to the COVID-19 epidemic and economic crisis*

Which of the things you just described would you say were influenced by COVID-19?

*Interviewer guidelines:*

- *If it feels more natural to clarify as the participant is answering the previous question, and if they were in the same arrangement before the pandemic as they are during the interview, you can ask them if the health or wellbeing issue they are describing was their experience before COVID-19 as well. But the question below should also be asked.*
- *If the participant focuses on the pandemic’s impact on their sense of health and well-being without mentioning work (lockdown, isolation, etc.), redirect specifically to ask how those feelings or conditions may be related to their employment.*

In what [other] ways, if any, might changes in your employment situation as a result of COVID-19 be related to your health and your family’s health in the day-to-day?

**Cope/deal with difficulties**

*Interviewer guidelines:*

- *Ideally issues about coping/dealing with difficulties have come up organically or have been asked above about impacts.*
- *Ways of coping or dealing with difficulties may be of any type or level*

We’ve talked about some difficulties that you have had because of your employment in general and specific to COVID-19. Are there any ways you have found to manage those difficulties or make them less impactful for you?

**Policies/practices that support health/wellbeing of workers and their families**

*Interviewer guidelines:*

- *Try to spend considerable time on the questions in this section.*
- *If participant’s talk is largely positive about their experiences/ health and well-being impacts, and if you feel asking any of the policy prompts below would force the issue, try to explore specifically what makes them feel supported. For instance, “What about the [e.g. local policies/ practices/ other people they mention] has been helpful to your own situation?”*

I’d like to ask you about things that exist to support the health and wellbeing of workers and their families. These might be things a specific employer does, or things governments (cities/provinces/states/countries) do to lessen the problems that can come for workers in these circumstances. What employment policies, if any, can you think of that were supportive of your health and well-being and that of your family?

I’d like to hear more about how (X thing the interviewee talked about) helps. Please tell me more about how it works for you and your family?

We talked about existing policies and practices that support your health or wellbeing. What policies or practices, if any, that do not exist yet could help you and your family?

Prompts

- Do you think politicians, unions etc. pay enough attention to the concerns of people in non-standard employment under normal circumstances? How so?
- Things that might come to mind could be:
- Limiting working hours
- Setting manageable expectations
- Encouraging workers not to email off-hours
- Allowing breaks to call home
- Inviting worker representation in decision-making groups
- Setting wages at a level people can live reasonably on
- Higher unemployment benefits if people lose their jobs (for a longer time)
- Free or subsidized childcare organized by the employer or the state (on-site, close to home)
- Universal basic income (including for people who do not work)
- [Insert country-specific prompts for general policies (not specific to COVID-19) as relevant and as identified in country context document]

*Clarify/probe about policies specific to the COVID-19 epidemic and economic crisis*

*Interviewer guidelines:*

- *If policies specific to COVID-19 have not already come up, begin by saying something like the following:*

We talked about policies that support workers and their families in general. Now I would like to hear about policies specific to the COVID-19 crisis. What employment policies, if any, can you think of that were supportive of your health and well-being under pandemic conditions?

I’d like to hear more about how (X thing the interviewee talked about) helps. Please tell me more about how it works for you?

What policies or practices, if any, that do not exist yet could help you under pandemic conditions?

Prompts specific to policies because of the coronavirus pandemic

- Exceptional extended paid sick COVID -19 leave
- Exceptional extra health insurance/healthcare policies
- Exceptional housing policies (halting evictions and electricity and water shutoffs, freezing rent and utility payments)
- Temporary delay of mortgage payments
- Working from home
- [Insert country-specific prompts for policies specific to COVID-19 as relevant and as identified in country context document]

**Closing**

- Is there anything else I have not asked you about that you would like to add?
- Inform the participant that you will stop the audio recording
- Overall feeling about the interview?
  - You can ask this if it feels natural in flow of the interview. Write down anything you feel is relevant in the memo afterwards.
- Can we contact you again if something is unclear or in case of further questions?
- Updates about the research (e.g. new article): project-website in informed consent form
- Contact interviewer/researcher (e.g. about results of the research): contact details in informed consent form
- Word of thanks + incentive/present
- After the interview: use the memo template to make notes about your overall impression of the interview and some contextual characteristics that might be important (e.g. about the home of the interviewee)

**Reference**

1. Creswell JW, Poth CN. Qualitative inquiry and research design: choosing among five approaches. Los Angeles: Sage Publications; 2017.
