# Supplementary material for: Labour and social protection gaps impacting the health and well-being of workers in non-standard employment: An international comparative study
Source: PLoS One. 2025 Mar 25;20(3):e0320248. doi: 10.1371/journal.pone.0320248 (PMC11936240; doi:10.1371/journal.pone.0320248)
Supplement: S2 File — Detailed description of the qualitative analysis (PDF) [file pone.0320248.s004.pdf]

Manuscript title: Labour and social protection gaps impacting the health and well-being of workers in non-standard employment: An international comparative study

Supportive material S2 File. Analytical strategy of multi-country individual worker data

Phases	Actions	All country teams	Single country teams
1. Single case analysis: codebook thematic analysis adapted from Braun & Clarke [1,2] Single case = country			
1a	<u>Deductive</u>: Developed the codebook		
i	Drew upon literature for dimensions and components related to precarious and NSE [3–5].	x	
	<u>Example codes & definitions</u> (edited for space constraints):<br>• *Degree of employment stability/security*: This code refers to the interviewee's perception of the degree of stability and security in their employment arrangement (i.e. temporariness in the contract, and threats to the continuation of employment).<br>• *Perception and views of experience of policies/support received:* This code refers to descriptions of actual experiences or opinions about receiving or attempting to enroll in programs/policies.	x	
ii	Held discussions with team members to reach consensus about code definitions.	x	
iii	Applied codes to one sample interview collaboratively.	x	
1b	<u>Inductive</u>: Captured emergent phenomena		x
i	Each site approached this at their discretion; example steps were: a) multiple team members read the interview transcripts, b) individual notes on new ideas for codes were generated, and, c) team discussions were held to confirm new codes and definitions.		x

	<u>Example codes & definitions</u> (edited for space constraints):<br><br>*Procuring work* (US)*:* This code refers to descriptions of the interviewee looking for work when it is integral to their occupation (e.g. freelance technician).<br>*Knowledge and understanding of employment conditions and rights* (SE): This code refers to descriptions of participants' lack of clarity about employment contracts, information, etc., and employers' unclear explanations about the same.		
1c	Transcribed all interviews and applied all codes in languages in which interviews were conducted		x
ii	Generated a complete codebook with codes, definitions, and exemplary quotes.		x
iii	Generation of country-specific themes		x
2. Preparation for cross-case analysis (multiple case study adapted from Stake, 2006)[6]			
i	Developed single case report template (adapted from Stake, 2006, p.15).	x	
ii	Wrote single-case reports.		x
iii	Developed single case evaluation template (adapted from Stake, 2006, p.45).	x	
3a. Cross-case analysis - Stage 1			
i	Paired country teams according to theorized differences in economic and welfare state typologies to exchange respective single case reports.	x	
ii	Evaluated the exchanged single case by using the single case evaluation template to summarize and assess most salient themes.		x
iii	Created a multi-case matrix with countries on one axis and study research questions in the other.	x	

iv	Populated salient themes and research question response narratives on the multi-case matrix that drew from the single case evaluation sheets after reaching consensus with paired country team.		x
v	Summarized similarities and differences across all cases according to study research questions using multi-case matrix.	x	
vi	Held discussions that led to the generation of additional tailored research questions addressed in this and forthcoming articles.	x	
3b. Cross-case analysis - Stage 2			
i	Created an additional cross-country matrix with the new research questions and populated it with salient themes from the first cross-country matrix.	x	
ii	Supplemented themes with, when necessary, additional reviews of each country case report, interview transcripts or analytical products from the first phase.		x
iii	Summarized similarities and differences across all cases.	x	

References

1. Braun V, Clarke V. Thematic Analysis. In: Cooper H, Camic P, Long D, Panter A, Rindskopf D, Sher K, editors. APA Handbook of Research Methods in Psychology, Vol 2: Research Designs: Quantitative, Qualitative, Neuropsychological, and Biological. Washington DC: American Psychological Association; 2012. pp. 57–71.

2. Braun V, Clarke V. Conceptual and design thinking for thematic analysis. Qual Psychol. 2022;9: 3–26. doi:10.1037/qup0000196

3. Bodin T, Çağlayan Ç, Garde AH, Gnesi M, Jonsson J, Kiran S, et al. Precarious employment in occupational health – an OMEGA-NET working group position paper. Scand J Work Environ Health. 2019. doi:10.5271/sjweh.3860

4. Kreshpaj B, Orellana C, Burström B, Davis L, Hemmingsson T, Johansson G, et al. What is precarious employment? A systematic review of definitions and operationalizations from

quantitative and qualitative studies. Scand J Work Environ Health. 2020. doi:10.5271/sjweh.3875

5. International Labour Office (ILO). Non-standard employment around the world: understanding challenges, shaping prospects. Geneva: International Labor Organization; 2016. Available: http://www.ilo.org/global/publications/books/WCMS_534326/lang--en/index.htm

6. Stake RE. Multiple case study analysis. New York, NY: The Guilford Press; 2006.
